# A Platelet-Powered Drug Delivery System for Enhancing Chemotherapy Efficacy for Liver Cancer Using the Trojan Horse Strategy

**DOI:** 10.3390/pharmaceutics16070905

**Published:** 2024-07-05

**Authors:** Hao Huang, Xiaoping Wang, Ziqing Gao, Hongyi Bao, Xiaopeng Yuan, Chao Chen, Donglin Xia, Xiangqian Wang

**Affiliations:** 1 Nantong Institute of Technology, Affiliated Tumor Hospital of Nantong University, School of Public Health of Nantong University, Nantong 226000, China; huanghao@ntit.edu.cn (H.H.); xpw77@ntu.edu.cn (X.W.); 2217320024@stmail.ntu.edu.cn (Z.G.); yuanxiaopeng198708@163.com (X.Y.); chenchao@ntu.edu.cn (C.C.); 2 Department of Laboratory Medicine, Affiliated Hospital of Nantong University, Medical School of Nantong University, Nantong 226001, China; 2331310106@stmail.ntu.edu.cn; 3 Radiotherapy Department of Nantong Tumor Hospital, Nantong 226361, China

**Keywords:** chemotherapy, tumor-targeted, accumulation, platelets, liver cancer

## Abstract

Optimizing the delivery and penetration of nano-sized drugs within liver cancer sites, along with remodeling the tumor microenvironment, is crucial for enhancing the efficacy of chemotherapeutic agents. For this study, a platelet (PLT)-mediated nanodrug delivery system (DASA+ATO@PLT) was developed to improve the effectiveness of chemotherapy. This system delivers nano-sized dasatinib and atovaquone specifically to liver tumor sites and facilitates intra-tumoral permeation upon release. Through JC-1, immunohistochemistry, and DNA damage analyses, the therapeutic effect of DASA+ATO@PLT was assessed. In vitro simulation and intravital imaging were carried out to determine the accumulation of dasatinib and atovaquone in liver tumor sites. The experiment demonstrated the accumulation of dasatinib and atovaquone in tumor sites, followed by deep permeation in the tumor microenvironment with the assistance of PLTs, while simultaneously revealing the ability of DASA+ATO@PLT to remodel the liver cancer microenvironment (overcoming hypoxia) and enhance chemotherapeutic efficacy. This system utilizes the natural tumor recognition ability of PLTs and enhances the chemo-immunotherapeutic effect through targeted delivery of nano-chemotherapeutic drugs to the tumor, resulting in effective accumulation and infiltration. The PLT-mediated nanodrug delivery system serves as a “Trojan horse” to carry therapeutic drugs as cargo and deliver them to target cells, leading to favorable outcomes.

## 1. Introduction

Liver cancer presents a significant threat to human health, due to its aggressive nature, rapid proliferation, and high incidence. Globally, it is the third most common cause of cancer-related death, claiming approximately 400,000 lives annually in China alone [1,2,3,4]. Chemotherapy is widely recognized as one of the most effective therapeutic approaches for cancer [5,6]; however, liver cancer is often diagnosed at advanced stages, characterized by a hostile tumor microenvironment with insufficient oxygen, inadequate blood flow, and limited nutrient availability [7,8]. This results in resistance to chemotherapy and reduces the efficacy of treatment. Moreover, most liver cancer patients experience impaired liver function, exacerbating the toxic adverse effects of chemotherapy. Addressing these challenges effectively requires enhancing drug accumulation and penetration at liver cancer sites, as well as remodeling of the tumor microenvironment.

The application of nano-chemotherapeutic drugs has demonstrated potential in improving drug penetration within tumor cells and inhibiting the growth of solid tumors [9,10,11]. However, the clinical implementation of nanomedicine in liver cancer treatment presents challenges. For instance, dasatinib (DASA), a third-generation tyrosine kinase inhibitor, has been demonstrated to have limited efficacy in clinical applications, even in its nano-sized formulation. Its low response rates, high resistance rates, and poor tolerability render it a less-viable option for the treatment of hepatocellular carcinoma [12,13,14]. Furthermore, its significant side effects and the common occurrence of liver function impairment among liver cancer patients pose obstacles to its application [15]. Consequently, researchers have shifted their focus towards enhancing the tumor microenvironment. Through hindering electron transfer in the coenzyme Q of the cell respiratory chain, they aim to decrease cellular respiration through inhibiting the synthesis of mitochondrial respiratory chain complexes I and III [16,17]. This process restricts cellular oxidative phosphorylation, ultimately enhancing the effectiveness of photodynamic therapy [18,19]. However, achieving the desired therapeutic effects via direct intravenous injection or oral administration of atovaquone (ATO) is challenging. In this context, utilizing targeted drug delivery mechanisms and increasing the local drug concentration can significantly enhance the efficacy of the drug [20,21].

The Trojan Horse drug delivery strategy utilizes carrier systems, such as nanoparticles or liposomes, to clandestinely transport drug molecules to specific targets, ultimately enhancing the efficacy of the drug while minimizing potential side effects [22,23]. This strategy aims to enhance drug bioavailability, target specificity, and therapeutic outcomes in various medical applications, including cancer treatment and the management of infectious diseases. Among Trojan Horse drug delivery strategies, as platelets (PLTs) possess the ability to evade phagocytosis by the endothelial system, prolong the drug’s presence in the bloodstream, and successfully target tumor sites by traversing biological barriers, they have been proven to be particularly effective carriers [24,25]. These inherent characteristics of PLTs have been harnessed to facilitate efficient and targeted drug delivery [26]. For instance, Hu et al. effectively employed platelet membranes to encapsulate polymeric nanoparticles, resulting in the targeted delivery of tumor necrosis factor and an anticancer drug [27]. This approach demonstrated a reduction in blood clearance, enhanced tumor-targeting efficiency, and inhibition of distant metastasis of cancer cells. PLTs used as carriers to develop a tumor-targeted drug delivery system have also been reported by Xia et al. [28]. This system alleviates tumor hypoxia through oxygenation, thereby enabling low-dose radiation therapy for cancer treatment.

**Scheme 1:**
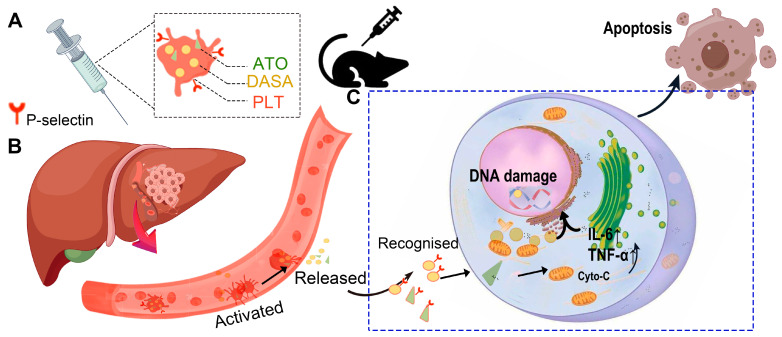
Schematic representation of the Trojan Horse strategy using DASA+ATO@PLT for liver cancer: (**A**) illustration of DASA+ATO@PLT; (**B**) DASA+ATO@PLTs recognizing tumor cells via P-selectin, leading to the targeted release of nano-DASA and ATO at the liver tumor site; and (**C**) the DASA+ATO@PLT system enhanced the effectiveness of chemotherapy and improved its efficacy mechanism.

Building upon the aforementioned Trojan Horse strategy, this study suggests employing PLTs as potent nanodrug carriers, with DASA serving as the representative drug, while additionally incorporating ATO as a chemosensitizer to augment the effectiveness of liver cancer therapy (Scheme 1). This approach was developed with the aim of establishing a novel PLT-powered nanodrug delivery system through encapsulating DASA and ATO within PLTs—designated as DASA+ATO@PLT (Scheme 1A)—thus mitigating the adverse toxic effects associated with chemotherapeutic drugs. Additionally, specific targeting of tumor behavior was achieved through the interaction between P-selectin and PLT surfaces, facilitating the effective delivery of drugs to liver tumor sites (Scheme 1B). The subsequent release of ATO promptly inhibited tumor cell respiration, thereby improving the liver cancer microenvironment. Consequently, this approach enhanced the effectiveness of chemotherapy and achieved the desired objectives of tumor treatment (Scheme 1C). Focusing on restructuring the tumor microenvironment to enhance the efficacy of chemotherapy, our findings represent a novel method for augmenting the antitumor effect of chemotherapy drugs.

## 2. Materials and Methods

### 2.1. Materials and Animals

Atovaquone (ATO, 1.35 g/cm^3^), DASA (MW ≈ 488), Prostaglandin E1 (PGE1) (MW ≈ 354.48), and adenosine diphosphate (ADP) (MW ≈ 427.2) were obtained from Bomei Biotechnology (Hefei, China). Dimethyl sulfoxide and dimethylformamide were purchased from J&K Scientific (Beijing, China). CCK8 solution, JC-1 kit, AM-PI, DAPI, Cy5.5, DAB, and other staining solutions were all obtained from Beyotime (Shanghai, China). HRP antibody, γ-H2AX antibody, Alexa Fluor 594-labeled goat anti-rabbit antibody, tumor necrosis factor-alpha (TNF-α), and interleukin-6 (IL-6) antibody were obtained from Boao Biological (Beijing, China). Further, the 7721 liver cancer cell line and HUVEC cell line were both purchased from Binglemon Biotechnology (Zhengzhou, China). The cells were at passage number 3 and used up to passage number 9.

Sprague Dawley (SD) rats (275–300 g) and BALB/c mice (15–20 g) were purchased from the Experimental Animal Center of Nantong University, China, and used under protocols approved by the aforementioned center for in vivo antitumor studies. Animals were housed at a controlled temperature of 20–22 °C, relative humidity of 40–70%, and 12 h/12 h light/dark cycles, and fed with standard laboratory chow and tap water ad libitum. All animal experiments were conducted in accordance with relevant ethical regulations and institutional guidelines provided by the Division of Comparative Medicine at Nantong University.

The experimental procedures were approved by the Animal Research Committee of Nantong University (approval number: 20220922-329). They adhered to the European Parliament Directive 2010/63/EU on animal welfare and the guidelines set forth by the Division of Comparative Medicine of Nantong University (S202307061-023). All procedures were conducted in compliance with local regulations. Written informed consent was obtained from the animal owners for their participation in the study. Live animal imaging was performed under anesthesia (2% isoflurane in oxygen, using the XGI-8 XENOGEN Gas Anesthesia System, Longmed group Co., Ltd., Suphanburi, Thailand), according to the manufacturer’s instructions. In other studies, subcutaneous injections and tail vein injections were the only invasive procedures, which were considered less painful and, therefore, did not require anesthesia. At the conclusion of the experiments, mice were euthanized via 100% CO_2_ anesthesia.

### 2.2. Preparation of DASA+ATO@PLT

PLTs were prepared through heart blood sampling of SD rats under anesthesia. The blood was centrifuged at 200 g/min for 10 min and the supernatant was collected. The same procedure was repeated until the supernatant was clear of blood. Subsequently, PGE1 was added, followed by centrifugation at 1800 g/min for 20 min. The extracted PLTs were then dispersed in PBS and stored at room temperature.

Next, 75 mg of ATO was measured and dissolved in 1 mL of dimethyl sulfoxide to prepare the ATO solution. Then, 75 mg of DASA was dissolved in 1 mL of dimethylformamide to prepare the DASA solution [29,30]. The ATO solution, DASA solution, and PLTs were mixed in a 1:1:8 (*v*/*v*/*v*) ratio. The mixture was stirred for 5 min and sonicated under 100 W power for 10 min to prepare the DASA+ATO@PLT solution. DASA+ATO@PLT was then centrifuged at 1800 g/min for a further 5 min to remove the residue, and allowed to stand at room temperature.

### 2.3. Characterization of DASA+ATO@PLT

Next, 1 mL of 0.2 mg of the generated particles was suspended in 0.1 M sodium phosphate buffer (pH 7.0). Zeta cells were pre-equilibrated at 21 °C with the buffer. Three independent measurements were recorded for each of 12 runs from every batch. The generated data were fitted using the Smoluchowski approximation assuming Henry’s function f(Ka) of 1.5 [31]. To determine the particle size and Zeta potential of DASA solution, ATO solution, PLTs, and DASA+ATO@PLTs, they were each dispersed in a Zetasizer Nano ZS (Malvern, Worcestershire, UK). Particle size measurements were conducted using dynamic light scattering (DLS). The particle size in the DASA+ATO@PLT suspension was re-measured within 48 h to evaluate its stability.

The encapsulation rate was the total quality of DASA+ATO@PLTs after centrifuging the DASA+ATO@PLT mixture and absorbing the supernatant. The formula for calculating the encapsulation efficiency was the quality of packed DASA+ATO/total quality of DASA+ATO@PLTs × 100%. Drug loading efficiency (%) was calculated as (quality of loaded DASA+ATO/quality of added DASA+ATO) × 100%.

Following activation by ADP, DASA+ATO@PLTs were centrifuged at 1800× *g* for 10 min. The supernatant was collected to determine the peak UV absorption. The absorbance of DASA was measured at 325 nm using a Varian Cary 5000 UV-Vis-NIR spectrophotometer (Varian, California, USA). The release rate of DASA+ATO@PLTs was calculated as Release rate (%) = Da/(Da + Db) × 100%, where Da is the absorbance of DASA in the supernatant and Db is the absorbance of DASA in the precipitate. The precipitate was pre-treated with deionized water to cause the platelet microcarriers to rupture, thus releasing DASA. The mixture was centrifuged, DASA was collected, and the DASA concentration was measured at 325 nm. The absorbance of the ATO solution was detected at 241 nm. The morphological characteristics of DASA+ATO@PLT and its activated and released drugs were determined through scanning electron microscopy (JSM-6510, Tokyo, Japan). Active DASA+ATO@PLT treatments were performed as follows: after 10 μM of ADP was added, the DASA+ATO@PLTs were incubated at 37 °C. Samples were collected before ADP addition, as well as at 10 min and 2 h after ADP addition. The collected samples were treated with 2.5% (*v*/*v*) glutaraldehyde in PBS (0.1 M, pH 7.4) at 4 °C for 3 h. After the samples were washed with distilled water at room temperature and sprayed with gold, SEM images were obtained.

The biocompatibility of DASA+ATO@PLT was assessed via CCK8 assay. The vascular epithelial cells (HUVECs) were cultured at 37 °C with 5% CO_2_, and divided into four groups: phosphate-buffered saline (PBS), DASA, DASA+ATO, and DASA+ATO@PLT. Subsequently, 100 μL of the corresponding solution was added in each group and incubated for 24 h. After incubation, the culture medium was discarded and 10 μL of CCK8 solution (10 mg/mL) and 190 μL of cell culture medium were added to each well. Cells were incubated in the dark for 2 h and the absorbance was then measured at 450 nm using a spectrophotometer to calculate cell inhibition.

### 2.4. Western Blot Analysis (WB)

WB was performed using conventional WB procedures. CD62p proteins were extracted from PLTs, DASA+ATO@PLT, and activated DASA+ATO@PLT with ADP (molecular weight: 110 kDa) through Western blotting, with β-actin (molecular weight: 42 kDa) used as an internal reference protein (standard). The dilution ratio of CD62p was 1:1000, and that of β-actin was also 1:1000. The secondary antibody was mouse HRP (rabbit anti-mouse IgG, H&L), which was used at a dilution of 1:4000. A total of 60 μL of the resultant buffered solution was used for sodium dodecyl sulfate–polyacrylamide gel electrophoresis (SDS-PAGE) on a 10% SDS-PAGE gel at 100 V. The proteins were then transferred using a semi-dry transfer method to a polyvinylidene difluoride (PVDF) membrane, followed by bathing in 5% skimmed milk (20 mL) for 2 h. Key platelet membrane proteins were identified using a CD62p antibody. The membranes were stored overnight at 4 °C and then washed with 50 mL of PBS containing 0.05% *v*/*v* Tween 20 before the addition of rabbit anti-mouse HRP-conjugated secondary antibodies and incubation for another 2 h at room temperature. The membranes were combined with 1 mL of the ECL luminescent reagent followed by scanning (Epson Perfection V370 Photo, Seiko Epson Corporation, Suwo, Japan) and imaging using the ImageJ software (National Institutes of Health, ImageJ V1.8.0, Bethesda, MD, USA) in order to quantify the amount of proteins present.

### 2.5. Changes in Mitochondrial Membrane Potential

Log-phase 7721 liver cancer cells were collected and seeded in 6-well plates at 6 × 10^5^ cells per well, followed by incubation for 24 h in a 5% carbon dioxide incubator at 37 °C. Subsequently, the cells were treated with 200 µL of PBS, DASA, DASA+ATO, or DA-SA+ATO@PLT for 48 h. After treatment, the cells were washed with PBS, followed by the addition of 500 µL of culture medium and 500 µL of the JC-1 staining working solution (5×). The mixture was thoroughly mixed and incubated for 20 min. Cells were washed with pre-cooled JC-1 wash solution (1×) twice before adding 2 mL of cell culture medium. The fluorescence of the JC-1 monomer (green) and the aggregated one (red) was measured using confocal laser scanning microscopy (FV 3000, Olympus, Center Valley, PA, USA) with excitation/emission settings at 514 nm/585 nm.

The average fluorescence intensity allows for semi-quantitative analysis of specific protein expression. It yields a single-channel (monochrome) fluorescence image, where the gray value of each pixel represents the fluorescence intensity at that point. ImageJ was used to detect the average fluorescence intensity: Mean gray value (Mean) = Integrated Density (IntDen)/The Area (Area).

### 2.6. Cell Apoptosis Assay

Log-phase 7721 liver cancer cells were counted and plated in a 6-well plate at 6 × 10^5^ cells per well, followed by incubation for 24 h in a 5% carbon dioxide incubator at 37 °C. The cells were then treated with 200 µL of PBS, DASA, DASA+ATO, or DASA+ATO@PLT for another 24 h. After two washes with PBS, 5 µL of 4 mM Calcein-AM solution and 5 µL of 16 mM PI solution were added to each well. The wells were incubated at room temperature for 30 min, washed twice with PBS, and observed under a fluorescence microscope (495 nm/530 nm) for photography. The Calcein AM stain exhibited green fluorescence, suggesting that the cells remained viable and non-toxic. Conversely, propidium iodide (PI) staining revealed red fluorescence, indicating that the drug combination was capable of inducing apoptosis.

### 2.7. DNA Damage Assessment

DNA damage in cells was assessed using phosphorylated histone γ-H2AX. The 7721 liver cancer cells were plated in a 24-well plate at 1 × 10^5^ cells per well, followed by incubation for 24 h in a 5% carbon dioxide incubator at 37 °C. The cells were then treated with 200 µL of PBS, DASA, DASA+ATO, or DASA+ATO@PLT, and further incubated for 24 h. Following fixation with paraformaldehyde and washing with PBS, the cells were incubated with Triton-X 100 at room temperature for 15 min and then blocked with goat serum. After another wash with PBS, a phosphorylated histone γ-H2AX rabbit polyclonal antibody (1:500) was added, and the mixture was incubated overnight at 4 °C. This was followed by washing again, then an Alexa Fluor 594-labeled goat anti-rabbit secondary antibody was added and the solution was incubated in the dark at room temperature for 2 h. The cells were again washed with PBS, stained with DAPI, and sealed with an anti-fade mounting medium. The formation of γ-H2AX foci was then observed under a fluorescence microscope (594 nm).

### 2.8. Establishment of the Model

When the 7721 liver cancer cells reached 90% confluence, they were harvested and adjusted to a concentration of 3 × 10^7^ cells/mL. Subcutaneous injections of 100 µL of the cell suspension were administered into the upper right thigh of male BALB/c mice. The model was deemed successfully established when the solid tumor size reached 100 mm^3^.

### 2.9. In Vivo Imaging

Biodistributions of the samples were monitored using the IVIS Lumina III in vivo imaging system (Perkin Elmer, Waltham, MA, USA). Before injection, the fluorescent dye Cy5.5 was activated with EDC and NHS. Then, nano-DASA+ATO was labeled with Cy5.5 at a concentration of 1 mg/mL. Tumor-bearing mice were intravenously injected with 100 µL of Cy5.5-labeled DASA+ATO and DASA+ATO@PLT solutions. The distribution and intensity of fluorescence were monitored 20 min post-injection using an animal imaging system (stage temperature: 37 °C, excitation wavelength: 648 nm, emission wavelength: 662 nm, with a 1 s exposure).

### 2.10. Evaluation of Therapeutic Efficacy

The 40 mice were randomly allocated into four groups, with 10 mice in each group. Tail vein injections with 0.1 mL of PBS, DASA, DASA+ATO, or DASA+ATO@PLT were administered. Injections were administered every 3 d, for a total of four treatments. The body weights of the mice were recorded, and tumor volumes were measured every morning at 9 a.m. using calipers. Tumor volume was calculated using the following formula: V = 0.5 ab^2^, where ‘a’ represents the longest diameter and ‘b’ represents the shortest diameter. The survival rates and changes in tumor size were noted. At 4 d post-final injection, the mice were euthanized, and the tumor and the major organs such as the heart, liver, spleen, lungs, and kidneys were harvested for examination. Hematoxylin and eosin (H&E) staining was performed on the collected tissues.

Paraffin-embedded tissue sections underwent antigen retrieval and blocking with BSA, and were incubated overnight at 4 °C with tumor necrosis factor-alpha (TNF-α) and interleukin-6 (IL-6) antibodies. Following treatment with a fluorescent secondary antibody, the sections were stained with DAPI in the dark, washed, and sealed with an anti-fade mounting medium. Fluorescence imaging was performed under a fluorescence microscope.

### 2.11. TUNEL Staining

The tumor tissues were paraffin-embedded, sectioned, and washed. Proteinase K was applied, followed by incubation for 10 min at 37 °C. The TUNEL staining solution was then added, with a further hour of incubation at 37 °C. After rinsing, DAB was applied for a 10 min reaction in the dark, followed by hematoxylin staining for 5 min. The sections were differentiated in hydrochloric acid–methanol for 5 s, dehydrated in a graded series of alcohol, and sealed. Tumor cell apoptosis was observed and imaged under an optical microscope. Biotin-labeled dUTP binds to the 3′-OH terminal of broken DNA under the action of deoxyribonucleotide terminal transferase, then binds specifically to streptavidin of horseradish catalase and produces a strong color response in the presence of diaminobenidine, the substrate of horseradish catalase. Dark brown particles are DNA of apoptotic cells.

### 2.12. Inflammatory Reaction Test

After subcutaneous injections of PBS, DASA, DASA+ATO, or DASA+ATO@PLT were administered, blood was drawn every 5 days. Blood examinations revealed that the total number of white blood cells (WBCs), neutrophils (NEs), lymphocytes (LYs), and monocytes (MOs) remained within the normal range after DASA+ATO@PLT treatment. During the treatment, liver function indices such as alkaline phosphatase (ALP), alanine aminotransferase (ALT), and aspartate aminotransferase (AST), as well as renal function indices such as blood urea nitrogen (BUN), were also assessed. The HEMAVET 950FS Hematology System Analyzer was used for hematological assays. Biochemical analyses were performed using an automated biochemical analyzer (Trilogy, Eaubonne, France).

### 2.13. Statistical Analysis

All values were averaged and are expressed as mean ± standard error of the mean. Multiple group comparisons were evaluated through ANOVA, whereas t-tests were used to evaluate the significance between two groups. A value of *p* < 0.05 was considered statistically significant.

## 3. Results

### 3.1. Construction and Characterization of DASA+ATO@PLT

**Figure 1:**
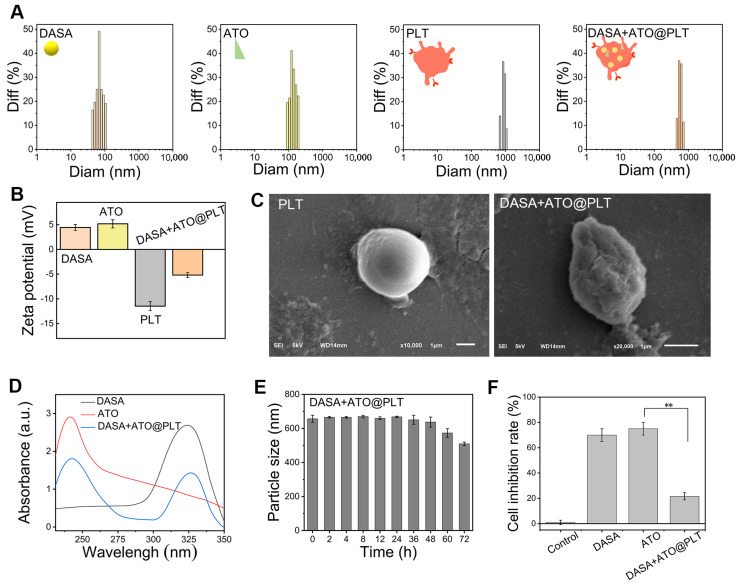
Characterization of PLT-based nanodrug delivery system (DASA+ATO@PLT): (**A**) size distribution of DASA, ATO, PLTs, and DASA+ATO@PLT; (**B**) Zeta potential of DASA, ATO, PLTs, and DASA+ATO@PLT; (**C**) SEM characterization results of resting PLTs and DASA+ATO@PLT; (**D**) UV-vis spectra of DASA, ATO, and DASA+ATO@PLT; (**E**) stability of DASA+ATO@PLT in PBS in vitro; and (**F**) cell inhibition rate results of DASA+ATO@PLT. ** *p* < 0.01.

A PLT-based nanodrug delivery system was constructed with the aim of treating liver cancer, called DASA+ATO@PLT, which is shown in Figure 1. The DASA and ATO particles were approximately 100 nm in size, as illustrated in Figure 1A. In contrast, the PLT carriers had a diameter of 850 ± 60 nm, which was sufficient to facilitate encapsulation of the DASA and ATO. After encapsulation, the particle size of DASA+ATO@PLT was 615 ± 84 nm. The change in particle size distribution indicated that the PLT drug carrier did not break during the drug loading process, and remained in a resting state after drug loading [32]. Based on the results shown in Figure 1B, the surface potential of DASA+ATO@PLT differed from the positive charge of DASA and ATO, displaying a negative potential akin to PLTs. To enhance the drug loading capacity of PLTs, PEG1 was employed to suppress PLT activation during drug loading. PEG1 inhibits the aggregation of PLTs by binding to the PLT surface glycoprotein Ib (GPIb) and preventing it from interacting with the von Willebrand factor (VWF), which is essential for PLT aggregation. Activated PLTs would aggregate and release their contents, which directly leads to changes in their morphology and particle size. Therefore, the changes in morphology and particle size during loading of PLTs with DASA and ATO were investigated in this study, in order to evaluate the effect of PEG1 on maintaining the resting state of PLTs [33,34]. The surface morphology of DASA+ATO@PLT is presented in Figure 1C, wherein the DASA+ATO@PLT morphology closely resembles that of resting PLTs with a diameter of approximately 650 nm, corroborating the findings of the particle size analysis. UV spectrum results (Figure 1D) revealed significant absorption peaks at 241 nm and 325 nm, corresponding to ATO (241 nm) and DASA (325 nm), respectively. The presence of these absorption peaks in DASA+ATO@PLT confirmed the successful encapsulation of DASA and ATO within the PLTs. To minimize drug costs, the optimal loading conditions were investigated. We found that the loading content was associated with the amount of DASA (Figure S1). When the DASA concentration increased to 7.5 mg/mL, the drug loading of DASA+ATO@PLTs attained the best economic benefits. The loading efficiency of DASA in DASA+ATO@PLTs reached 38.7 ± 2.5%, even when it was negatively associated with the initial concentration (Figure S2). Thus, in the following experiments, this optimized dose (7.5 mg/mL) was adopted to achieve high loading content without compromising the DASA loading efficiency. Overall, these findings demonstrate that PLT could successfully envelop DASA and ATO, completing construction of the DASA+ATO@PLT. The stability of DASA+ATO@PLT in PBS was assessed, focusing on particle size variation as a key indicator of stability. As shown in Figure 1E, DASA+ATO@PLT maintained a stable particle size (660 ± 23 nm) over 48 h, indicating the formulation’s favorable in vitro storage properties. In addition, we sought to improve the biocompatibility of DASA and ATO after encapsulation in PLT. The in vitro cytotoxicity assessment of DASA+ATO@PLT (Figure 1F) indicated that the normal HEK cell inhibition rate was significantly reduced after inclusion of nano-DASA and ATO in PLT, indicating the enhanced biocompatibility of DASA+ATO@PLT.

### 3.2. Liver Tumor—Responsive Targeting and Release Behaviors of DASA+ATO@PLT

**Figure 2:**
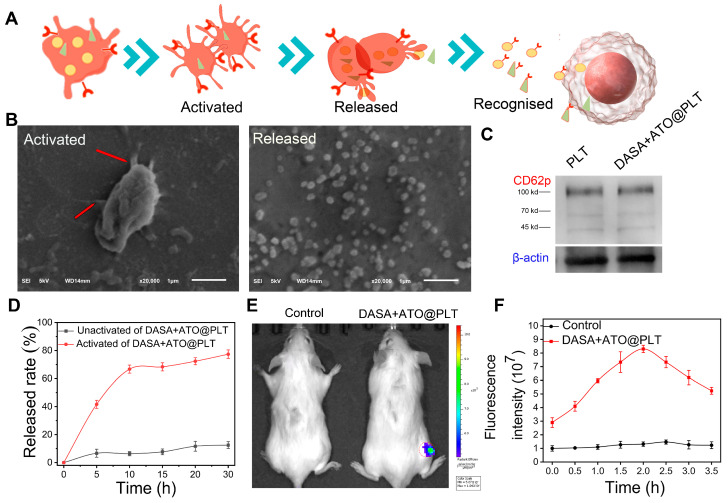
Targeted tumor delivery effects of DASA+ATO@PLT: (**A**) schematic illustration of tumor targeting and release process; (**B**) SEM images of DASA+ATO@PLT during the activation process and fully activated DASA+ATO@PLT. The red arrows refer to pseudopodia in activated DASA+ATO@PLT; (**C**) P-selectin bands (CD62p, 110 kDa) of pristine PLTs and activating DASA+ATO@PLT in Western blotting. β-actin (42 kDa) was used as the internal reference protein (standard); (**D**) DASA release time curve of DASA+ATO@PLT after activation; (**E**) live imaging at 2 h after administration; and (**F**) change curve of fluorescence intensity in live imaging at the tumor site after administration of DASA+ATO@PLT.

In this study, DASA+ATO@PLT was designed to activate, aggregate, and release its contents upon stimulation by tumor-related factors within the tumor vasculature. The released DASA and ATO present platelet-specific P-selectin on their surfaces, enabling targeted recognition and delivery to HCC cells within the tumor microenvironment, as shown in Figure 2A. Based on this, we first investigated the activation of DASA+ATO@PLT. A key factor for the success of this strategy is the ability of liver cancer cells to activate DASA+ATO@PLT and trigger drug release. Previous studies have shown that ADP interacts with P2Y1 and P2Y12 receptors on PLT surfaces, initiating a cascade of signal transduction pathways which, in turn, affect the intracellular and extracellular calcium levels, protein kinase activities, and integrin ligand-binding functions, thus altering PLT shape, stability, and granule release [35,36]. Unlike normal cells, malignant tumor cells could spontaneously form and release ADP, inducing PLT activation and drug release [37]. Therefore, ADP was used in our study to simulate the activation of DASA+ATO@PLT in vitro, with SEM employed to monitor this process. The SEM results in Figure 2B show the DASA+ATO@PLT during the activation process after ADP incubation. At the beginning, PLTs can be observed extending pseudopodia and undergoing morphological changes. After 2 h, DASA+ATO@PLT was fully activated, gradually releasing the encapsulated DASA and ATO. The released DASA+ATO@PLT appeared as regular spherical particles approximately 200 nm in diameter, consistent with particle size analysis results (Figure S3), showing a diameter of 172 ± 30 nm. P-selectin is an essential component for PLTs to recognize and adhere to tumor cells [38]. The WB results clearly indicated the presence of P-selectin (110 kDa) in DASA+ATO@PLT (Figure 2C), revealing that P-selectin was still on the surface of activating DASA+ATO@PLT. Post-activation, DASA was released from DASA+ATO@PLT with an initial rapid release, while the final release rate at 10 h was 77.3 ± 3.2%, as shown in Figure 2D. In contrast, DASA+ATO@PLT not activated by ADP showed a minimal release (release rate ≈ 10%). The release curve of DASA+ATO within 10 h conformed to the linear model with an R^2^ of 0.99296, indicating zero-order drug release kinetics (Figure S4). Subsequently, the DASA+ATO release rate gradually decreased.

To further explore the targeted delivery of DASA+ATO@PLT in vivo, Cy5.5-labeled DASA+ATO@PLTs were employed. Two hours after tail vein injection in tumor-bearing mice, the DASA+ATO@PLT group exhibited pronounced fluorescence at the tumor site, as shown in Figure 2E. The fluorescence intensity within the tumor tissue was then analyzed, as shown in Figure 2F. The fluorescence intensity in DASA+ATO@PLT increased over time, peaking at 2 h post-administration.

The in vitro cellular experiments and animal studies collectively demonstrated that DASA+ATO@PLT could effectively target liver cancer tissues, releasing the encapsulated drugs and enhancing drug concentration within the liver cancer tissues.

### 3.3. Remodeling of the Tumor Microenvironment by DASA+ATO@PLT

**Figure 3:**
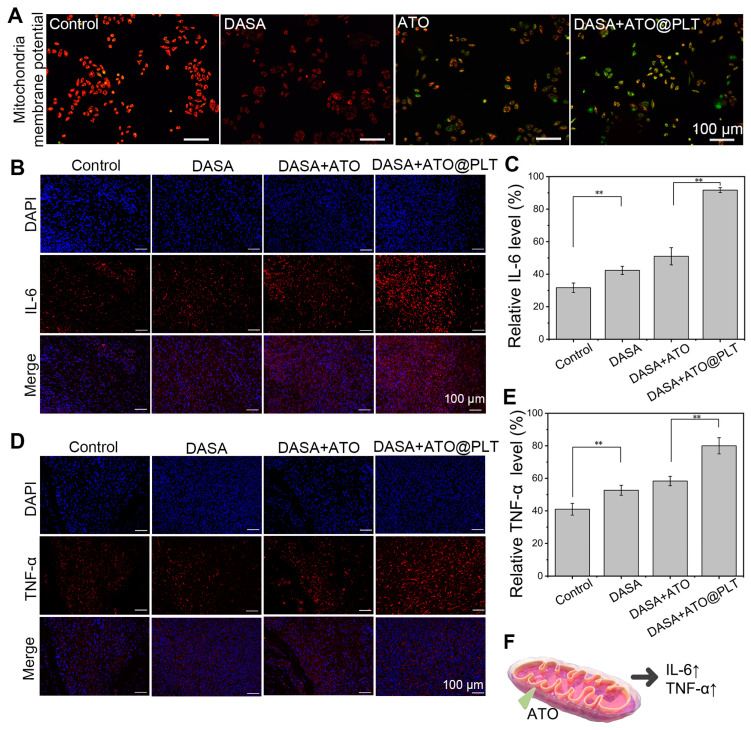
Remodeled tumor microenvironment results of DASA+ATO@PLT: (**A**) the changes in mitochondrial membrane potential in vitro as detected by JC-1. Red: Normal membrane potential; Green: Mitochondrial membrane potential depolarization; (**B**) fluorescence expression of pro-inflammatory cytokines IL-6 in vivo. DAPI: Blue; IL-6: Red; Merge: Blue + Red; (**C**) statistical analysis of IL-6 expression levels; (**D**) fluorescence expression of pro-inflammatory cytokines TNF-α in vivo. DAPI: Blue; IL-6: Red; Merge: Blue + Red; (**E**) statistical analysis of TNF-α expression level; and (**F**) schematic illustration of ATO suppressing mitochondrial function, inducing elevated levels of IL-6 and TNF-α. ** *p* < 0.01.

After targeting liver tumor cells, the ATO particles released from DASA+ATO@PLT. As a chemotherapy drug sensitizer, ATO can inhibit the electron transfer of respiratory chain coenzyme Q in liver cancer cells, reduce cell respiration, and inhibit the synthesis of mitochondrial respiratory chain complex I and complex III, thus inhibiting the oxidative phosphorylation process of cells, inhibiting the mitochondrial function of liver cancer cells, and reshaping the tumor microenvironment [16,17]. Then, the changes in the mitochondrial membrane potential in liver tumor cells were studied after treatment with DASA+ATO@PLT. The fluorescence probe JC-1 was used to detect the changes in mitochondrial membrane potential (Δψm). As shown in Figure 3A, the control group (PBS-treated) displayed strong red fluorescence, indicative of high mitochondrial membrane potential (normal condition). In contrast, the liver tumor cells treated with DASA+ATO@PLT exhibited green fluorescence, signaling a lowered mitochondrial membrane potential. This indicated that DASA+ATO@PLT altered the membrane potential of the tumor cells. The rate of membrane potential changes in the DASA+ATO@PLT group was significantly different from that of the control group (*p* < 0.01), as shown in Figure S5.

To further validate the efficacy of DASA+ATO@PLT in remodeling the tumor microenvironment in vivo, changes in the inflammatory cytokines, such as interleukin-6 (IL-6) and tumor necrosis factor-alpha (TNF-α), within the tumor were examined. As is well known, IL-6 can mediate inflammatory responses, whereas TNF-α is central to coordinating inflammatory immune responses, reflecting direct changes in the tumor microenvironment [39,40]. IL-6 and TNF-α were chosen as inflammatory indicators to evaluate the changes in the tumor microenvironment. As shown in Figure 3B,C, the relative expression rate of IL-6 in the control group was only 32.5 ± 2.9%, while DASA+ATO@PLT increased the expression rate of IL-6 to 92.3 ± 1.5%. There was a significant difference between DASA+ATO@PLT and the control group (*p* < 0.01). Furthermore, there was a large amount of red fluorescence (positive) after DASA+ATO@PLT treatment (80.2 ± 5.4%), suggesting that liver tumor cells secreted substantial amounts of TNF-α, indicating a serious inflammatory response as compared with the control group (*p* < 0.01; Figure 3D,E). The expression of TNF-α was significantly increased (*p* < 0.01). In contrast, the changes in inflammation were not as pronounced in the group treated solely with DASA, as ATO can inhibit the synthesis of mitochondrial respiratory chain complex I and III, resulting in cell respiration, thus leading to an improved tumor microenvironment and increased levels of inflammatory response factors, including IL-6 and TNF-α (Figure 3F). A slight improvement was observed in the DASA+ATO group, possibly as the inhibitory function of ATO was not effective at the tumor sites. This may be due to most of the drugs having been cleared by macrophages in the bloodstream or metabolized by the kidneys, thus failing to reach the tumor site directly. Therefore, the favorable inflammatory responses in the DASA+ATO@PLT group may be attributed to the excellent targeting ability of PLT.

### 3.4. Therapeutic Efficacy In Vivo

**Figure 4:**
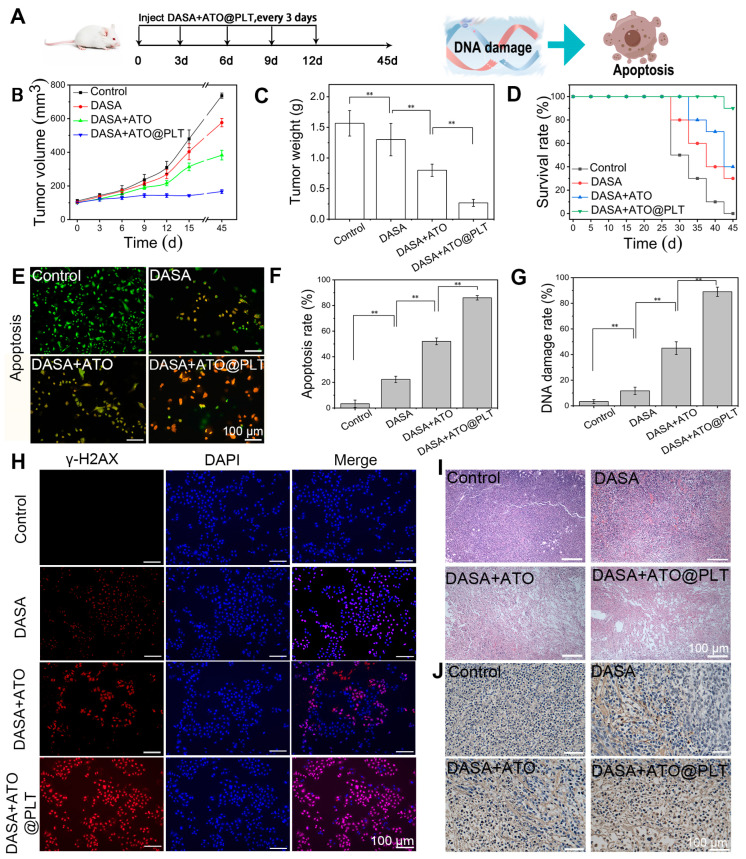
Therapeutic effects of DASA+ATO@PLT: (**A**) Administration diagram. The DASA+ATO@PLT was administered every 3 d for a total of five times; (**B**) tumor volume change curve during drug administration; (**C**) tumor quality in mice after 45 d of treatment; (**D**) survival curve during the 45 d treatment; (**E**) immunofluorescence microscopy results of apoptosis; (**F**) statistical analysis of cell apoptosis; (**G**) statistical analysis of DNA damage; (**H**) immunofluorescence of γ-H2AX. Red: γ-H2AX; Blue: DAPI nuclear staining; (**I**) H&E staining results of tumor tissue sections at 45 d post-treatment; and (**J**) TUNEL assay of tumor tissue sections at 45 d post-treatment. Scale, 100 μm. ** *p* < 0.01.

To validate its therapeutic effect in vivo, the chemotherapeutic impact of DASA+ATO@PLT was further studied. The drug administration scheme is depicted in Figure 4A. DASA+ATO@PLT was administered through tail vein injections every 3 d for a total of four doses. After 45 d, the volume and weight of tumors in the DASA+ATO@PLT-treated group were significantly suppressed (Figure 4B,C), compared to the control group (*p* < 0.01). Additionally, no excessive body weight loss was observed in the DASA+ATO@PLT-treated group (Figure S6). Tumor growth was slightly suppressed in groups treated solely with DASA or DASA+ATO, but the tumor volume gradually increased over time.

Furthermore, all mice in the control group (PBS-treated) succumbed to death within 45 d, whereas the survival rate in the DASA+ATO@PLT group reached 90%. Mice treated solely with DASA or DASA+ATO had survival rates of only 30% and 40%, respectively, as shown in Figure 4D. The comparatively lower survival rates in the DASA and DASA+ATO groups may be due to lower drug aggregation in the tumor. In contrast, a higher survival rate after DASA+ATO@PLT treatment was observed, which may be attributed to the specific binding of PLTs to tumor cells via the interaction of surface P-selectin with the hyaluronic acid receptor (CD44) expressed on tumor cells, enabling drugs to target the damaged areas [41]. This allowed for the efficient adhesion of DASA and ATO to the tumor cell surface, thus facilitating drug release and penetration, in the DASA+ATO@PLT group. The cell apoptosis rate in the DASA+ATO@PLT group was noted, while slight increases were observed in the DASA and DASA+ATO groups (Figure 4E,F). DASA treatment produces DNA damage through inducing DNA double-strand breaks, which could be assessed by detecting phosphorylated histone γ-H2AX. As shown in Figure 4G,H, the DASA+ATO@PLT group displayed abundant red fluorescent dots in the nucleus, with a mean fluorescence intensity of 89.5 ± 3.6%, much higher than that in the DASA group (11.7 ± 2.9%) and DASA+ATO group (45.2 ± 5.4%), indicating more serious DNA damage in cells treated with DASA+ATO@PLT. There were significant differences among all groups (*p* < 0.01). In contrast, almost no red fluorescence signals of γ-H2AX were observed in the control group, indicating no double-stranded DNA breaks. The pathological analysis of tumor tissues post-treatment is shown in Figure 4I, indicating that extensive necrosis occurred only in tumor tissues treated with DASA+ATO@PLT, whereas PBS caused almost no damage, and only slight damage was observed in the DASA or DASA+ATO groups. The H&E staining results corroborated the TUNEL assay findings (Figure 4J and Figure S7), showing a pronounced increase in apoptotic cells (brown granules) in the DASA+ATO@PLT group. There was a significantly stronger apoptosis-inducing effect in the DASA+ATO@PLT group than treatment with only DASA or DASA+ATO. These findings further illustrated that DASA+ATO@PLT enhanced the efficacy of the chemotherapeutic drug DASA in the liver tumors.

### 3.5. Safety Evaluation

**Figure 5:**
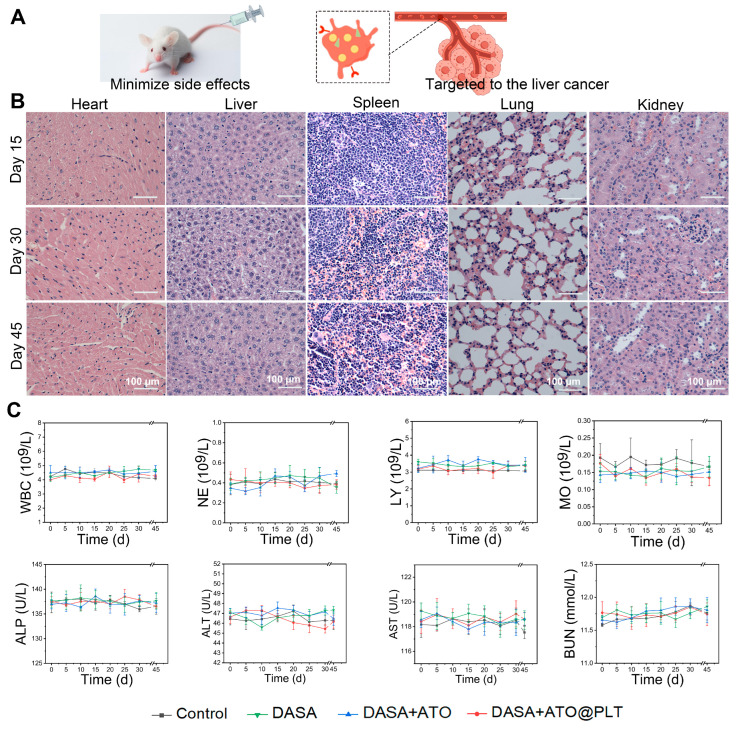
Safety evaluation of DASA+ATO@PLT: (**A**) schematic of the mechanism by which DASA+ATO@PLT minimizes toxic side effects; (**B**) H&E staining results of vital organs, including the heart, liver, spleen, lung, and kidney. Scale bar: 100 μm. (**C**) Measurement of biochemical blood indices, including white blood cell (WBC), neutrophil (NE), lymphocyte (LY), and monocyte (MO) counts and alkaline phosphatase (ALP), alanine aminotransferase (ALT), aspartate aminotransferase (AST), and urea nitrogen (BUN).

As a result of encapsulating DASA and ATO in PLTs, direct interactions with vital organs could be minimized, thereby potentially reducing toxic side effects (Figure 5A). After DASA+ATO@PLT treatment, the mice were euthanized and a safety assessment was performed. As shown in Figure 5B, there were no abnormal pathological changes in the heart, liver, spleen, lung, or kidney, and significant inflammatory cell aggregation or interstitial fibrosis was not observed in these major organs. The internal morphological structure of the cells was observed under H&E staining, and the cells were not deformed and had good morphology. Blood examinations revealed that the total number of white blood cells (WBCs), neutrophils (NEs), lymphocytes (LYs), and monocytes (MOs) remained within the normal range after DASA+ATO@PLT treatment (Figure 5C). During the treatment, liver function indices such as alkaline phosphatase (ALP), alanine aminotransferase (ALT), and aspartate aminotransferase (AST), as well as renal function indices such as blood urea nitrogen (BUN), were all in their normal ranges. This suggests no apparent hepatic or kidney dysfunction following DASA+ATO@PLT treatment. These results confirm that DASA+ATO@PLT possesses excellent biocompatibility.

## 4. Discussion

Traditional systemic chemotherapy has demonstrated some success in the clinical treatment of cancer; however, studies have shown that the liver tumor microenvironment can compromise the efficacy of chemotherapeutic agents [42,43,44,45]. Hence, enhancing the susceptibility of tumor tissue to chemotherapy presents a promising strategy to augment its effectiveness [46,47]. DASA inhibits the interaction between Src tyrosine kinase and hepatocyte growth factor receptor, enhancing the sensitivity of cancer cells to tumor necrosis factor-related apoptosis-inducing ligands [48,49,50]. A pH-sensitive small molecule developed by Tang et al., which involved conjugating DASA with an ortho-ester bond of an acid, demonstrated substantial antitumor efficacy through targeted drug delivery to cancer cells [51]. DASA has also been utilized to induce immunomodulation in solid tumors, resulting in reduced intra-tumoral T-cells and delayed tumor progression in melanoma, sarcoma, colorectal, and breast cancers [52,53].

Inhibiting mitochondrial respiration has been identified as a key strategy to enhance chemotherapy outcomes [54,55]. ATO was used as a chemotherapy sensitizer in this study, in order to boost the responsiveness of liver tumor cells to DASA through inhibiting mitochondrial function. Ashton et al. have reported that ATO at pharmacological concentrations significantly reduces the oxygen consumption rate of tumor cells, thus promoting a hypoxic environment inside tumor cells and reducing their energy supply, enhancing the efficacy of radiotherapy, and delaying tumor growth [56]. Similarly, ATO-treated tumor cells exhibited reduced oxygen consumption and ATP levels, enhancing tumor cell sensitivity. ATO can specifically target oxidative phosphorylation to inhibit cancer cell proliferation, making it a promising drug for ovarian cancer chemoprevention and chemotherapy [29,57]. This study indicated that using ATO as a chemosensitizer for tumor cells resulted in the inhibition of cellular respiration, improvement of the tumor microenvironment, enhanced sensitivity to lower concentrations of DASA, and reduced adverse effects.

Earlier studies necessitated increasing the dosage concentration to achieve the desired therapeutic effect [58,59]. Higher concentrations of DASA tend to induce auto-toxic side effects, such as a marked decrease in platelet count and bone marrow suppression. Consequently, targeted medication for liver cancer becomes crucial in precisely regulating the inhibition of tumor cell growth and proliferation. Post-modification with polyethylene glycol, drugs showed extended half-life and absorption time, enhancing the tumor targeting ability and immunogenicity [60]. However, this method can also induce the body to produce specific antibodies, hindering the uptake of anticancer drugs by tumor cells, as well as affecting the absorption rate and permeability of nanomedicines [61]. The design of a nanoparticle-based drug delivery system significantly enhances in-body drug targeting and controlled drug release [58]. Therefore, this study employed platelets (PLTs) as natural drug carriers—a widely used approach in recent years. PLTs are abundant in human blood, rapidly respond to infection and damaged tissue, and play a vital role in tumor treatment and immunotherapy, making them an innovative vehicle for targeted drug delivery [62,63,64]. PLTs specifically bind to hyaluronic acid receptors (CD44) expressed on tumor cells through surface P-selectin, enabling them to target damaged areas and penetrate tumors for therapeutic effect [65,66]. In this study, PLTs were used as nanomedicine carriers, with ATO as a chemotherapy sensitizer combined with DASA, in order to construct the PLT-loaded liver cancer drug release system DASA+ATO@PLT. This PLT-based targeting system offers high specificity, effective targeting, and strong enrichment permeability, compared with previously reported drug delivery systems. The released ATO rapidly inhibits tumor cell respiration, addressing tumor hypoxia and improving the liver cancer microenvironment. Although PLT drug delivery strategies have shown potential as targeted drug delivery systems, further exploration is required to optimize PLT-mediated drug delivery. Addressing these challenges can improve the effectiveness of PLT-based drug delivery strategies and promote further research in this promising field, greatly enhancing the sensitivity of liver cancer cells to chemotherapy and providing new avenues for the clinical treatment of liver cancer.

## 5. Conclusions

This study proposed a nanoparticle-based drug delivery system utilizing the Trojan Horse strategy to enhance the efficacy of chemotherapy in the context of liver cancer. By employing PLTs as targeted drug carriers and ATO as a chemotherapeutic drug sensitizer for DASA, the DASA+ATO@PLT system was successfully developed. This system enables targeted drug delivery to the tumor tissue site, releasing ATO and DASA for precise therapeutic action. The released drugs are continuously enriched and penetrate the tumor, with ATO inhibiting liver cancer cell respiration. The notable antitumor effects of DASA+ATO@PLT demonstrate that the PLT-mediated nanodrug delivery system functions as a “Trojan horse”, effectively delivering the therapeutic drugs to target cells. This innovative combination of drug carriers and nano-chemotherapy drugs represents a promising strategy to enhance the antitumor effect against liver cancer.

## Data Availability

The study data are contained within the article and Supplementary Materials.

## Supplementary Materials

The following supporting information can be downloaded at https://www.mdpi.com/article/10.3390/pharmaceutics16070905/s1: Figure S1: Relationship between initial concentration and encapsulation efficiency; Figure S2: Relationship between initial concentration and drug loading efficiency; Figure S3: Particle size distribution of fully activated DASA+ATO@PLT; Figure S4: Fitting curve of zero-order kinetic equation; Figure S5: Statistical analysis of mitochondrial membrane potential; Figure S6: Mice body weight changes over time; Figure S7: Statistical analysis of changes in TUNEL.

## References

[1] Wang Y., Yan Q., Fan C., Mo Y., Wang Y., Li X., Liao Q., Guo C., Li G., Zeng Z. (2023). Overview and countermeasures of cancer burden in China. Sci. China Life Sci..

[2] Shen H., Wang Z., Ren S., Wang W., Duan L., Zhu D., Zhang C., Duan Y. (2020). Prognostic biomarker MITD1 and its correlation with immune infiltrates in hepatocellular carcinoma (HCC). Int. Immunopharmacol..

[3] Yao Q., Chen W., Yu Y., Gao F., Zhou J., Wu J., Pan Q., Yang J., Zhou L., Yu J. (2023). Human Placental Mesenchymal Stem Cells Relieve Primary Sclerosing Cholangitis via Upregulation of TGR5 in Mdr2(-/-) Mice and Human Intrahepatic Cholangiocyte Organoid Models. Research.

[4] Yang G., Yan H., Tang Y., Yuan F., Cao M., Ren Y., Li Y., He Z., Su X., Yao Z. (2023). Advancements in understanding mechanisms of hepatocellular carcinoma radiosensitivity: A comprehensive review. Chinese J. Cancer Res..

[5] Seehawer M., Heinzmann F., D’Artista L., Harbig J., Roux P., Hoenicke L., Dang H., Klotz S., Robinson L., Doré G. (2018). Necroptosis microenvironment directs lineage commitment in liver cancer. Nature.

[6] Kritfuangfoo T., Rojanaporn D. (2024). Update on chemotherapy modalities for retinoblastoma: Progress and challenges. Asia-Pac. J. Ophthalmol..

[7] Bai R., Li Y., Jian L., Yang Y., Zhao L., Wei M. (2022). The hypoxia-driven crosstalk between tumor and tumor-associated macrophages: Mechanisms and clinical treatment strategies. Mol. Cancer.

[8] Zhao Q., Dong X.L., Zhu C.Y., Zhang Y., Fang C., Zhou X.L., Zhang K., Zhou H. (2023). DNA damage-encouraged Mn-As-based nanoreactors reshape intratumoral cell phenotypes to recover immune surveillance and potentiate anti-tumor immunity. Chem. Eng. J..

[9] Girigoswami A., Girigoswami K. (2023). Potential Applications of Nanoparticles in Improving the Outcome of Lung Cancer Treatment. Genes.

[10] Zheng S., Jiang F., Ge D., Tang J., Chen H., Yang J., Yao Y., Yan J., Qiu J., Yin Z. (2019). LncRNA SNHG3/miRNA-151a-3p/RAB22A axis regulates invasion and migration of osteosarcoma. Biomed. Pharmacother..

[11] Li L., He S., Liao B., Wang M., Lin H., Hu B., Lan X., Shu Z., Zhang C., Yu M. (2024). Orally Administrated Hydrogel Harnessing Intratumoral Microbiome and Microbiota-Related Immune Responses for Potentiated Colorectal Cancer Treatment. Research.

[12] Keating G.M. (2017). Dasatinib: A Review in Chronic Myeloid Leukaemia and Ph plus Acute Lymphoblastic Leukaemia. Drugs.

[13] Sesumi Y., Suda K., Mizuuchi H., Kobayashi Y., Sato K., Chiba M., Shimoji M., Tomizawa K., Takemoto T., Mitsudomi T. (2017). Effect of dasatinib on EMT-mediated-mechanism of resistance against EGFR inhibitors in lung cancer cells. Lung Cancer.

[14] Zeng X.L., Zhang Y.W., Xu X., Chen Z., Ma L.L., Wang Y.S., Guo X.L., Li J.C., Wang X. (2022). Construction of pH-sensitive targeted micelle system co-delivery with curcumin and dasatinib and evaluation of anti-liver cancer. Drug Deliv..

[15] Alanazi A., Alhazzani K., Alrewily S., Aljerian K., Algahtani M., Alqahtani Q., Haspula D., Alhamed A., Alqinyah M., Raish M. (2023). The Potential Protective Role of Naringenin against Dasatinib-Induced Hepatotoxicity. Pharmaceuticals.

[16] Xiang H., Tang H., He Q., Sun J., Yang Y., Kong L., Wang Y. (2024). NDUFA8 is transcriptionally regulated by EP300/H3K27ac and promotes mitochondrial respiration to support proliferation and inhibit apoptosis in cervical cancer. Biochem. Bioph. Res. Commun..

[17] Zhang H., Zhai X., Liu Y., Xia Z., Xia T., Du G., Zhou H., Strohmer D.F., Bazhin A.V., Li Z. (2023). NOP2-mediated m5C Modification of c-Myc in an EIF3A-Dependent Manner to Reprogram Glucose Metabolism and Promote Hepatocellular Carcinoma Progression. Research.

[18] Fu C., Xiao X., Xu H., Lu W., Wang Y. (2020). Efficacy of atovaquone on EpCAMCD44 HCT-116 human colon cancer stem cells under hypoxia. Exp. Ther. Med..

[19] Coates J., Skwarski M., Higgins G. (2019). Targeting tumour hypoxia: Shifting focus from oxygen supply to demand. Br. J. Radiol..

[20] Zhong C., Yang J., Zhang Y., Fan X., Fan Y., Hua N., Li D., Jin S., Li Y., Chen P. (2023). TRPM2 Mediates Hepatic Ischemia-Reperfusion Injury via Ca(2+)-Induced Mitochondrial Lipid Peroxidation through Increasing ALOX12 Expression. Research.

[21] Jha A., Kumar M., Bharti K., Manjit M., Mishra B. (2024). Biopolymer-based tumor microenvironment-responsive nanomedicine for targeted cancer therapy. Nanomedicine.

[22] Wu M., Zhang H., Tie C., Yan C., Deng Z., Wan Q., Liu X., Yan F., Zheng H. (2018). MR imaging tracking of inflammation-activatable engineered neutrophils for targeted therapy of surgically treated glioma. Nat. Commun..

[23] Ouyang X., Wang X., Kraatz H.B., Ahmadi S., Gao J., Lv Y., Sun X., Huang Y. (2019). A Trojan horse biomimetic delivery strategy using mesenchymal stem cells for PDT/PTT therapy against lung melanoma metastasis. Biomater. Sci..

[24] Yang H., Song Y., Chen J., Pang Z., Zhang N., Cao J., Wang Q., Li Q., Zhang F., Dai Y. (2020). Platelet Membrane-Coated Nanoparticles Target Sclerotic Aortic Valves in ApoE Mice by Multiple Binding Mechanisms Under Pathological Shear Stress. Int. J. Nanomed..

[25] Xiao G.Z., Zhang Z.K., Chen Q.Y., Wu T., Shi W., Gan L., Liu X.L., Huang Y., Lv M.Y., Zhao Y.X. (2022). Platelets for cancer treatment and drug delivery. Clin. Transl. Oncol..

[26] Mayorova O.A., Gusliakova O.I., Prikhozhdenko E.S., Verkhovskii R.A., Bratashov D.N. (2023). Magnetic Platelets as a Platform for Drug Delivery and Cell Trapping. Pharmaceutics.

[27] Hu Q., Sun W., Qian C., Wang C., Bomba H., Gu Z. (2015). Anticancer Platelet-Mimicking Nanovehicles. Adv. Mater..

[28] Xia D., Hang D., Li Y., Jiang W., Zhu J., Ding Y., Gu H., Hu Y. (2020). Au-Hemoglobin Loaded Platelet Alleviating Tumor Hypoxia and Enhancing the Radiotherapy Effect with Low-Dose X-ray. ACS Nano.

[29] Gonzalo R.-B., Rathi P., Nicole M., Alessandro B., Remko P., Martin M., Francesca M.B., Kevin J.H., Geoff S.H. (2024). Antitumour effect of the mitochondrial complex III inhibitor Atovaquone in combination with anti-PD-L1 therapy in mouse cancer models. Cell Death Dis..

[30] Khorshid M., Varshosaz J., Rostami M., Haghiralsadat F., Akbari V., Khorshid P. (2023). Anti HER-2 aptamer functionalized gold nanoparticles of dasatinib for targeted chemo-radiotherapy in breast cancer cells. Biomater. Adv..

[31] Tan X., He F.Y., Shang Y.B., Yin W.Z. (2016). Flotation behavior and adsorption mechanism of (1-hydroxy-2-methyl-2-octenyl) phosphonic acid to cassiterite. Trans. Nonferrous Met. Soc. China.

[32] Xu P.P., Zuo H.Q., Chen B., Wang R.J., Ahmed A., Hu Y., Ouyang J. (2017). Doxorubicin-loaded platelets as a smart drug delivery system: An improved therapy for lymphoma. Sci. Rep..

[33] Li S.P., Lu Z.F., Wu S.Y., Chu T.J., Li B.Z., Qi F.L., Zhao Y.L., Nie G.J. (2024). The dynamic role of platelets in cancer progression and their therapeutic implications. Nat. Rev. Cancer.

[34] Hashimoto H., Sugawara M., Tsuda H., Hirose S. (2009). Lipo PEG1 Therapy for Vascular Disturbances in SLE. Jpn. J. Clin. Immunol..

[35] Cattaneo M., Cerletti C., Harrison P., Hayward C.P.M., Kenny D., Nugent D., Nurden P., Rao A.K., Schmaier A.H., Watson S.P. (2013). Recommendations for the standardization of light transmission aggregometry: A consensus of the working party from the platelet physiology subcommittee of SSC/ISTH. J. Thromb. Haemost..

[36] Emmanuel Boadi A., Philomena E., Samara A., Glenn P.D., Satya P.K., Laurie E.K., Elisabetta L. (2022). Sex-related differences in the response of anti-platelet drug therapies targeting purinergic signaling pathways in sepsis. Front. Immunol..

[37] Bambace N.M., Levis J.E., Holmes C.E. (2010). The effect of P2Y-mediated platelet activation on the release of VEGF and endostatin from platelets. Platelets.

[38] Qi C.L., Wei B., Zhou W.J., Yang Y., Li B., Guo S.M., Li J.L., Ye J., Li J.C., Zhang Q.Q. (2015). P-selectin-mediated platelet adhesion promotes tumor growth. Oncotarget.

[39] Subedi L., Lee S.E., Madiha S., Gaire B.P., Jin M., Yumnam S., Kim S.Y. (2020). Phytochemicals against TNFα-Mediated Neuroinflammatory Diseases. Int. J. Mol. Sci..

[40] Wang Y.X., Zhou Y., Lin H., Chen H.Y., Wang S. (2022). Paeoniflorin Inhibits the Proliferation and Metastasis of Ulcerative Colitis-Associated Colon Cancer by Targeting EGFL7. J. Oncol..

[41] Chu P.Y., Tsai S.C., Ko H.Y., Wu C.C., Lin Y.H. (2019). Co-Delivery of Natural Compounds with a Dual-Targeted Nanoparticle Delivery System for Improving Synergistic Therapy in an Orthotopic Tumor Model. ACS Appl. Mater. Interfaces.

[42] Wang G., Li J.L., Bojmar L., Chen H.Y., Li Z., Tobias G.C., Hu M.Y., Homan E.A., Lucotti S., Zhao F.B. (2023). Tumour extracellular vesicles and particles induce liver metabolic dysfunction. Nature.

[43] Liu G.X., Wen Z.F., Liu F.Y., Xu Y.Q., Li H.J., Sun S.G. (2023). Multisubcellular organelle-targeting nanoparticle for synergistic chemotherapy and photodynamic/photothermal tumor therapy. Nanomedicine.

[44] Qi F., Li J., Qi Z., Zhang J., Zhou B., Yang B., Qin W., Cui W., Xia J. (2023). Comprehensive Metabolic Profiling and Genome-wide Analysis Reveal Therapeutic Modalities for Hepatocellular Carcinoma. Research.

[45] Zhang C., Jing X., Guo L., Cui C., Hou X., Zuo T., Liu J., Shi J., Liu X., Zuo X. (2021). Remote Photothermal Control of DNA Origami Assembly in Cellular Environments. Nano Lett..

[46] Zhang J., Song Q., Wu M., Zheng W. (2021). The Emerging Roles of Exosomes in the Chemoresistance of Hepatocellular Carcinoma. Curr. Med. Chem..

[47] Xia D., Zhang X., Hao H., Jiang W., Chen C., Li H., Feng L., Li J., Wu Y., Zhang L. (2023). Strategies to prolong drug retention in solid tumors by aggregating Endo-CMC nanoparticles. J. Control. Release.

[48] Dong Y., Liang C., Zhang B., Ma J., He X., Chen S., Zhang X., Chen W. (2015). Bortezomib enhances the therapeutic efficacy of dasatinib by promoting c-KIT internalization-induced apoptosis in gastrointestinal stromal tumor cells. Cancer Lett..

[49] Li W., Wang Z., Su Q., Chen J., Wu Q., Sun X., Zhu S., Li X., Wei H., Zeng J. (2024). A Reconfigurable DNA Framework Nanotube-Assisted Antiangiogenic Therapy. JACS Au.

[50] Zhang C., Yuan Y., Wu K., Wang Y., Zhu S., Shi J., Wang L., Li Q., Zuo X., Fan C. (2021). Driving DNA Origami Assembly with a Terahertz Wave. Nano Lett..

[51] Gao J., Qiao Z., Liu S., Xu J., Wang S., Yang X., Wang X., Tang R. (2021). A small molecule nanodrug consisting of pH-sensitive ortho ester-dasatinib conjugate for cancer therapy. Eur. J. Pharm. Biopharm..

[52] Hekim C., Ilander M., Yan J., Michaud E., Smykla R., Vähä-Koskela M., Savola P., Tähtinen S., Saikko L., Hemminki A. (2017). Dasatinib Changes Immune Cell Profiles Concomitant with Reduced Tumor Growth in Several Murine Solid Tumor Models. Cancer Immunol. Res..

[53] Sara A.E., Julia M.D., Lukas S., Moritz P., Ute N., Uwe K., Giuliano C. (2024). Interaction of the chemotherapeutic agent oxaliplatin and the tyrosine kinase inhibitor dasatinib with the organic cation transporter 2. Arch. Toxicol..

[54] Li W., Xiao X., Qi Y., Lin X., Hu H., Shi M., Zhou M., Jiang W., Liu L., Chen K. (2023). Host-Defense-Peptide-Mimicking β-Peptide Polymer Acting as a Dual-Modal Antibacterial Agent by Interfering Quorum Sensing and Killing Individual Bacteria Simultaneously. Research.

[55] Meng H., Yu Y., Xie E., Wu Q., Yin X., Zhao B., Min J., Wang F. (2023). Hepatic HDAC3 Regulates Systemic Iron Homeostasis and Ferroptosis via the Hippo Signaling Pathway. Research.

[56] Ashton T., Fokas E., Kunz-Schughart L., Folkes L., Anbalagan S., Huether M., Kelly C., Pirovano G., Buffa F., Hammond E. (2016). The anti-malarial atovaquone increases radiosensitivity by alleviating tumour hypoxia. Nat. Commun..

[57] Kapur A., Mehta P., Simmons A., Ericksen S., Mehta G., Palecek S., Felder M., Stenerson Z., Nayak A., Dominguez J. (2022). Atovaquone: An Inhibitor of Oxidative Phosphorylation as Studied in Gynecologic Cancers. Cancers.

[58] Wang D., Gao C., Zhou C., Lin Z., He Q. (2020). Leukocyte Membrane-Coated Liquid Metal Nanoswimmers for Actively Targeted Delivery and Synergistic Chemophotothermal Therapy. Research.

[59] Huang W., Pang Y.Z., Liu Q.F., Liang C.Y., An S.X., Wu Q.Y., Zhang Y., Huang G., Chen H.J., Liu J.J. (2023). Development and Characterization of Novel FAP-Targeted Theranostic Pairs: A Bench-to-Bedside Study. Research.

[60] Becker R., Dembek C., White L., Garrison L. (2012). The cost offsets and cost-effectiveness associated with pegylated drugs: A review of the literature. Expert Rev. Pharmacoecon. Outcomes Res..

[61] Wang H., Wu J., Williams G., Fan Q., Niu S., Wu J., Xie X., Zhu L. (2019). Platelet-membrane-biomimetic nanoparticles for targeted antitumor drug delivery. J. Nanobiotechnol..

[62] Przygodzki T., Talar M., Kassassir H., Mateuszuk L., Musial J., Watala C. (2018). Enhanced adhesion of blood platelets to intact endothelium of mesenteric vascular bed in mice with streptozotocin-induced diabetes is mediated by an up-regulated endothelial surface deposition of VWF-In vivo study. Platelets.

[63] Chen L., Zhou Z., Hu C., Maitz M.F., Yang L., Luo R., Wang Y. (2022). Platelet Membrane-Coated Nanocarriers Targeting Plaques to Deliver Anti-CD47 Antibody for Atherosclerotic Therapy. Research.

[64] Wang L., Gao H., Sun H., Ji Y., Song L., Jia L., Wang C., Li C., Zhang D., Xu Y. (2023). Reconfigurable Vortex-like Paramagnetic Nanoparticle Swarm with Upstream Motility and High Body-length Ratio Velocity. Research.

[65] Pattni B., Chupin V., Torchilin V. (2015). New Developments in Liposomal Drug Delivery. Chem. Rev..

[66] Ma Y., Zhang Y., Han R., Li Y., Zhai Y., Qian Z., Gu Y., Li S. (2022). A cascade synergetic strategy induced by photothermal effect based on platelet exosome nanoparticles for tumor therapy. Biomaterials.

