# Supplementary material for: A Platelet-Powered Drug Delivery System for Enhancing Chemotherapy Efficacy for Liver Cancer Using the Trojan Horse Strategy
Source: Pharmaceutics. 2024 Jul 5;16(7):905. doi: 10.3390/pharmaceutics16070905 (PMC11279470; doi:10.3390/pharmaceutics16070905)
Supplement: Supplementary file 1 [file pharmaceutics-16-00905-s001.zip › pharmaceutics-3064949-supplementary.pdf]

Supporting Information

A Platelet-Powered Drug Delivery System for Enhancing Chemotherapy Efficacy for Liver Cancer using the Trojan Horse Strategy

Hao Huang [1,†], Xiaoping Wang [1,†], Ziqing Gao [1], Hongyi Bao [2], Xiaopeng Yuan [1,3], Chao Chen [1], Donglin Xia [1,*] and Xiangqian Wang [1,3,*]

[1] Nantong Institute of Technology, Affiliated Tumor Hospital of Nantong University, School of Public Health of Nantong University. Nantong, 226000, China
[2] Department of Laboratory Medicine, Affiliated Hospital of Nantong University, Medical School of Nantong University, Nantong 226001, China
[3] Radiotherapy Department of Nantong Tumor Hospital, Nantong, 226361, China

* Correspondence:
Corresponding Author, xiadonglin@ntu.edu.cn (DL Xia); wangxiangqian@ntu.edu.cn (X Wang)

† These authors have contributed equally to this work

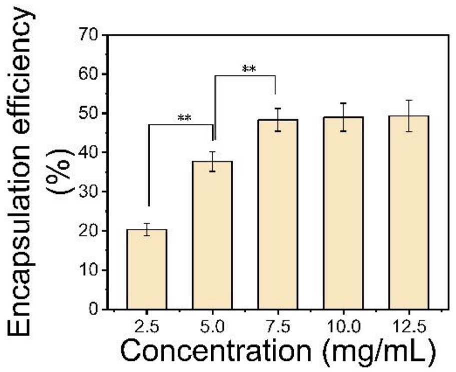

Figure S1. Relationship between initial concentration and encapsulation efficiency.

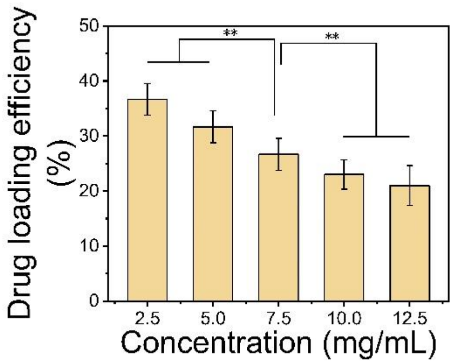

Figure S2. Relationship between initial concentration and drug loading efficiency.

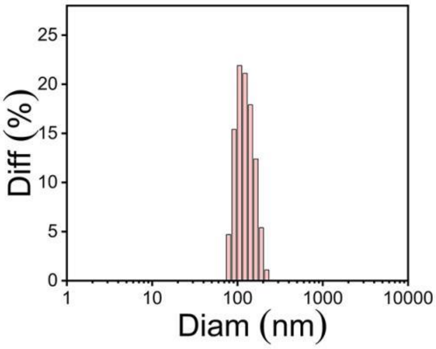

Figure S3. Particle size distribution of fully activated DASA+ATO@PLT.

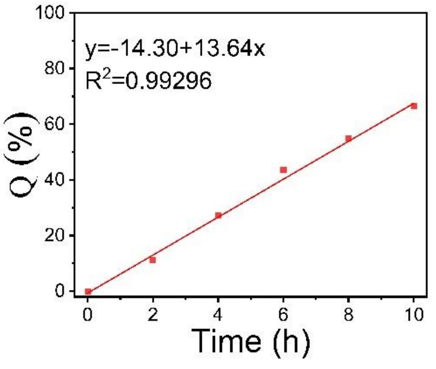

Figure S4. Fitting curve of zero-order kinetic equation.

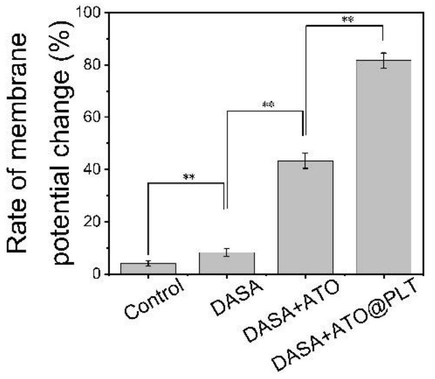

Figure S5. Statistical analysis of mitochondrial membrane potential.

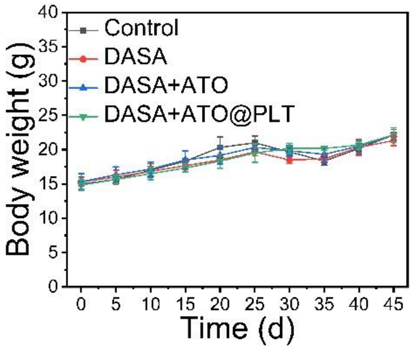

Figure S6. Mice body weight changes over time.

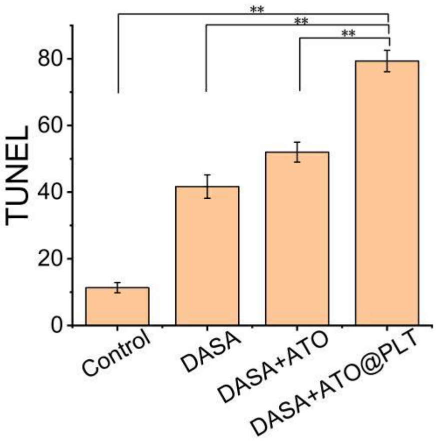

Figure S7. Statistical analysis of changes in TUNEL.
